# Integrins and the Metastasis-like Dissemination of Acute Lymphoblastic Leukemia to the Central Nervous System

**DOI:** 10.3390/cancers15092504

**Published:** 2023-04-27

**Authors:** Signe Modvig, Jenani Jeyakumar, Hanne Vibeke Marquart, Claus Christensen

**Affiliations:** 1 Department of Clinical Immunology, Copenhagen University Hospital Rigshospitalet, 2100 Copenhagen, Denmark; 2 Department of Clinical Medicine, Faculty of Health and Medical Sciences, University of Copenhagen, 2100 Copenhagen, Denmark

**Keywords:** integrin, acute lymphoblastic leukemia, immune surveillance, CNS, metastasis

## Abstract

**Simple Summary:**

The central nervous system constitutes a unique microenvironment to which access is highly restricted. However, immune cells enter as part of their normal routine surveillance, and some cancers, including leukemia in children, also spread to this site constituting a major challenge to therapy. Because of the restricted nature of the central nervous system, these cells are expected to make use of a defined set of adhesion molecules that will allow entry and residence. Integrins are a family of adhesion molecules, studied for decades in both normal immune cells and cancer cells. Here, we scrutinize the available knowledge to see if the same integrins are used by different cell types entering the central nervous system. By highlighting similarities between dissimilar cells and identifying gaps in our current understanding, the present review could be a helpful resource of ideas for future cancer research.

**Abstract:**

Acute lymphoblastic leukemia (ALL) disseminates with high prevalence to the central nervous system (CNS) in a process resembling aspects of the CNS surveillance of normal immune cells as well as aspects of brain metastasis from solid cancers. Importantly, inside the CNS, the ALL blasts are typically confined within the cerebrospinal fluid (CSF)-filled cavities of the subarachnoid space, which they use as a sanctuary protected from both chemotherapy and immune cells. At present, high cumulative doses of intrathecal chemotherapy are administered to patients, but this is associated with neurotoxicity and CNS relapse still occurs. Thus, it is imperative to identify markers and novel therapy targets specific to CNS ALL. Integrins represent a family of adhesion molecules involved in cell-cell and cell-matrix interactions, implicated in the adhesion and migration of metastatic cancer cells, normal immune cells, and leukemic blasts. The ability of integrins to also facilitate cell-adhesion mediated drug resistance, combined with recent discoveries of integrin-dependent routes of leukemic cells into the CNS, have sparked a renewed interest in integrins as markers and therapeutic targets in CNS leukemia. Here, we review the roles of integrins in CNS surveillance by normal lymphocytes, dissemination to the CNS by ALL cells, and brain metastasis from solid cancers. Furthermore, we discuss whether ALL dissemination to the CNS abides by known hallmarks of metastasis, and the potential roles of integrins in this context.

## 1. Introduction

Acute lymphoblastic leukemia (ALL) is the most frequently diagnosed form of cancer among children and adolescents with a five-year survival rate exceeding 90% [1,2]. Although the survival rate is high, treatment-related mortality also remains high and relapse occurs in 10–15% of the patients [3]. ALL is primarily a malignancy of the bone marrow and blood, but other hematopoietic and lymphatic tissues may also be involved including the spleen, thymus and lymph nodes [4]. Furthermore, ALL cells are seen in non-hematopoietic and non-lymphatic tissues, primarily the CNS, but also in the testis, skin, eyes, bones, breasts, muscles and abdominal organs [5,6]. Involvement of the CNS occurs with particularly high frequency, both at the time of diagnosis (8–13%) and upon relapse (10–30%) [7,8,9,10,11]. Contemporary treatment protocols use an early administered combination of intrathecal and high-dose systemic chemotherapy in the attempt to eradicate leukemic cells that potentially exist within the CNS at the time of diagnosis as well as prevent CNS relapse. However, neurotoxic adverse events occur in 4–12% of children in the form of seizures, stroke-like syndrome, posterior reversible encephalopathy syndrome or long-term neurocognitive deficits [9]. In addition, although alternative techniques are being explored [12,13,14,15], the conventional CNS staging of leukemia patients is still based on the microscopic examination of centrifuged CSF samples, which is associated with low sensitivity and poor specificity [13,16]. For these reasons, a call has been made for a paradigm shift in the treatment of CNS involvement, which includes more robust biomarkers, novel therapeutic targets and ways to overcome the barriers of the CNS microenvironment [9].

Leukemic cells develop from lymphoblasts, which themselves are destined to become circulating immune cells equipped with the abilities to home to, invade, and survey tissues as part of their normal immune functions. Notably, part of the normal function of leukocytes is the surveillance of the CNS including the passage across CNS barriers [17]. As such, leukemic cells can be viewed as preprogrammed for CNS dissemination perhaps explaining the extraordinary efficiency of ALL in accomplishing this process. In contrast, in solid cancers, metastasis designates the spread of cancer cells from their initial site to distant organs. It is conceived as an inefficient process with less than 0,01% of cells that enter circulation giving rise to metastasis [18,19]. For a solid, non-CNS cancer to metastasize to the CNS, the metastatic cells require capabilities in terms of invasion, survival in circulation, homing to the brain, adherence to and extravasation from blood vessels, as well as invasion and adaption to the brain microenvironment as either dormant or proliferating cells [20]. Decades ago, cancer cells were thought to require several genetic alterations to successfully go through all of these steps, referred to as the metastatic cascade [21]. However, current thinking explains metastasis as the result of specific driver oncogene mutations and conditions that maintain cancer cell self-renewal and plasticity. Rather than viewing metastasis as the accumulation of a series of cell-autonomous traits, the current view on metastasis emphasizes the role of cancer stem cells (CSCs) as well as the role of stromal cells and inflammation in generating metastatic niches sustaining the growth of metastasis [22,23,24].

The biological similarities and molecular aspects shared between solid cancer metastasis, the homing of immune cells and the spread of leukemia have occupied cancer scientists for decades and continues to do so [21,25,26]. Integrins represent a family of receptors binding cell surface and matrix ligands, which are acclaimed for their vital functions in adhesion and migration of normal cells and infamous for their role in the migration, adhesion and chemoresistance in malignant cells. Some of the same integrins would be expected to be involved in normal immune surveillance of the CNS, dissemination of ALL to the CNS and metastasis to the brain [27]. The important function of integrins in cell-adhesion-mediated chemoresistance has been covered elsewhere [28,29]. Here, we review the specific role of integrins in the spread of ALL to the CNS. Following a general introduction to integrins in immune cell migration and adhesion as well as CNS barriers and entry points, we specifically address how integrins are used by normal lymphocytes, ALL cells and solid cancer metastatic cells to gain access to the CNS. Finally, we address some of the contemporary hallmarks of metastasis, discussing whether ALL dissemination to the CNS conform to these hallmarks and the implications of integrins herein.

## 2. Integrins in Adhesion and Migration

**Figure 1:**
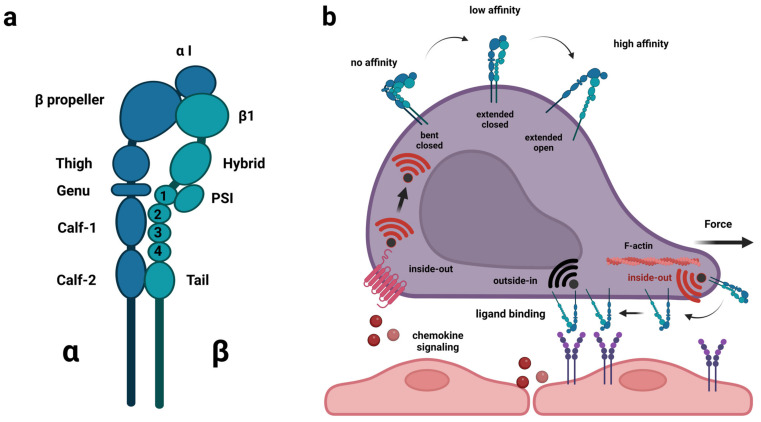
Structure of integrin heterodimers and regulation of integrin affinity on the cell surface. (**a**) heterodimer composed of α and β subunit. Shown is LFA-1 (α_L_β_2_). Integrin α-subunits expressed in leukocytes (α_L_, α_M_, α_D_ and α_X_) as well as α_1_, α_2_, α_10_ and α_11_ contain the so-called insertion or interaction domain (αI), which plays a vital part in ligand-binding of these integrins. All other α-subunits bind ligands in conjunction with the β-subunit, i.e., the head piece. (**b**) integrin heterodimers shift between bent conformation with closed head piece (no affinity for ligand), extended conformation with closed head piece (intermediate affinity) and extended conformation with open head piece (high affinity). Chemokine signaling and forces from the inside (e.g., actin-dynamics) can cause a shift between the conformations (inside-out activation), whereas binding to ligands or external forces (e.g., blood flow) can cause outside-in activation. This figure was created with BioRender.com (accessed on 21 April 2023).

The integrin gene family encodes alpha and beta proteins that are part of heterodimeric receptors (Figure 1a), which transduce signals in response to a range of diverse extracellular ligands as well as mechanical forces [30,31]. In humans, 18 alpha-subunits and 8 beta-subunits have been identified. They assemble into 24 different heterodimers, of which half have known functions in immune cells [32,33]. In terms of ligand specificity, integrin heterodimers are exceptionally versatile and redundant. A single integrin heterodimer, α_4_β_1_ (VLA-4), can bind the extracellular matrix molecules (ECM) fibronectin, thrombospondin, and osteopontin as well as the cell surface molecules vascular cell adhesion molecule 1 (VCAM-1) and mucosal addressin cell adhesion molecule 1 (MadCAM-1). With the exception of thrombospondin, the same ligand preferences are reported for α_4_β_7_. Conversely, depending on cell type and context, several integrin heterodimers bind laminin (α_6_β_4_ and β_1_ in complex with α_1_, α_2_, α_3_, α_6_, α_7_, α_10_ or α_V_) whereas a series of others (α_X_β_2_, and β_1_ in complex with α_1_, α_2_, α_10_ or α_11_) bind collagen [32]. Some of the major integrins found on immune cells are β_2_ in complex with α_L_ (LFA-1) or α_M_ (Mac-1) binding intracellular cell adhesion molecules (ICAMs) and various ECM molecules, but also α_4_β1 and α_4_β_7_ [33].

Although integrins are implicated in a wide range of biological processes, their direct role is commonly associated with cellular adhesion or migration. Integrins are ideally suited for this role as they can both sense outside molecules and mechanical forces while maintaining a physical binding to the intracellular machinery, underlying cellular morphology during rest and locomotion [34]. Hence, integrin proteins comprise a large ectodomain, a transmembrane domain and an intracellular domain. The α and β subunits fold to form a headpiece, which is responsible for ligand binding and can be either open or closed, and in the case of beta subunits, the intracellular domain contacts actin filaments via the proteins talin and kindlin [31,35]. Furthermore, integrins respond to bidirectional signaling, i.e., reacting to signals from both inside and outside the cell, and they shift between conformations associated with different levels of activation [30]. In the bent and closed conformation, the ligand affinity is low, in the extended closed configuration the ligand-affinity is intermediate whereas, in the extended open conformation, full activation has been achieved associated with strong ligand affinity [36]. During “inside-out” signaling, signals from other receptors or tensile forces within a cell cause the activation of integrins [37,38]. During migration, the actin polymerization at the leading edge creates tension within the integrin β-subunit due to the actin-integrin link via talin and kindlin, and this force induces an active integrin conformational state [39]. Additionally, during the adhesion and transendothelial migration of lymphocytes, chemokine signaling causes activation of integrin and this form of chemokine signaling contributes to the integrin ligand specificity dictating tissue-specific homing of lymphocytes [40,41]. Conversely, during “outside-in” signaling, ligand-binding or external mechanical forces affecting the integrin, cause the activation [31]. Of note, outside mechanical forces like blood or CSF flow are sufficient to induce so-called catch-bonds, which prolong the bond lifetime and provide more stable adhesion to ligands already engaged by integrins [42] (Figure 1b). Finally, in cancer cells, β integrin subunits are known to interact with a variety of growth factor receptors including the hepatocyte growth factor receptor c-Met, vascular endothelial growth factor receptor-2 (VEGFR-2), epidermal growth factor receptor (EGFR) and the ErbB2 receptor [43,44,45,46,47,48,49,50]. Through these interactions, the activation status of the integrins and the receptor tyrosine kinases (RTKs) are mutually changed. In the case of c-Met interaction with integrin β1, both outside-in and inside-out signaling take place [51], in addition to signaling from internalized integrin β1:c-Met complexes existing on the surface of endosomes, referred to as “inside-in” signaling [52]. The latter type of signaling occurs independently of integrin ligands and constitutes a way for cancer cells to sustain c-Met activation facilitating anchorage-independent survival and metastasis [46,52].

**Figure 2:**
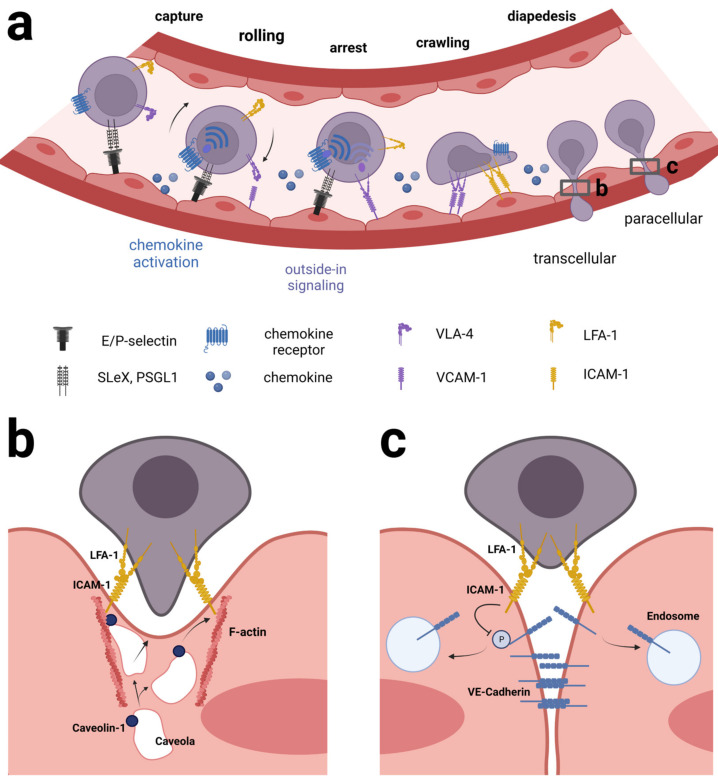
Mechanism of leukocyte extravasation from blood vessels. (**a**) The process starts by capture/tethering, which mainly depends on endothelial E-and P-selectins binding sialyl-Le X ligands or P-selectin glycoprotein ligands on the leukocyte surface, leading to leukocyte rolling on the luminal surface of the endothelium. VLA-4 (α_4_β_1_) binding to VCAM-1 and LFA-1 (α_L_β_2_) binding to ICAM-1 contribute to slowing down the rolling. A combination of chemokine activation and VCAM-1 and ICAM-1 binding increases the affinity of the integrins via inside-out and outside-in signaling, respectively. Additionally, the blood flow and the formation of catch bonds contribute to strengthening the LFA-1: ICAM-1 interaction. Eventually, this leads to arrest of the leukocyte. Following the formation of an LFA-1: ICAM-1 transmigratory cup, the leukocyte starts crawling and at the same time extends membrane protrusions to probe the endothelial surface to find a site permissive of transmigration. The last step of extravasation is diapedesis during which the leukocyte moves through the endothelium either through an individual EC cell (transcellularly) or through a space between neighboring EC (paracellularly) highlighted in (**b**,**c**), respectively. (**b**) transcellular diapedesis involves clusters of ICAM-1 on the endothelial cell, allegedly binding LFA-1 on the leukocyte. ICAM-1 is in physical contact with both F-actin filaments and the protein caveolin-1 on the surface of caveolae inside the endothelial cell. The caveolae gradually fuse to form a channel through which the leukocyte slides through. (**c**) In paracellular diapedesis, LFA-1 on the surface of the leukocyte binds ICAM-1 on two neighboring endothelial cells. ICAM-1 alters the phosphorylation status of key tyrosine residues in vascular-endothelial cadherin (VE-cadherin) causing VE-cadherin to be endocytosed by the endothelial cells. As a result, the adherens junctions between the endothelial cells are gradually unzipped as the leukocyte moves through. See text for further details. This figure was created with BioRender.com.

In leukocytes, integrins are used in a well-orchestrated way together with chemokine receptor signaling to achieve extravasation from blood across the endothelial cell wall and into inflamed tissue. The process of extravasation is believed to implicate the same series of consecutive steps and families of adhesion molecules in both CNS and non-CNS-vessels, yet the exact identity of adhesion molecules and chemokine receptors differ according to organ site [41,53,54]. Initially, the leukocyte and endothelial cells (EC) make contact in a process known as capture/tethering and rolling relying primarily on carbohydrate ligands on the leukocytes, e.g., PSGL-1 and selectins on the EC, e.g., P-selectin [55]. At this point, integrins have been known also to participate in the rolling process, e.g., α_4_β_7_ on leukocytes binding MadCAM1 on the EC [56]. Subsequently, during the activation step, the presence of chemokines becomes important because of the inside-out signaling they exert on integrins via G-protein coupled chemokine receptors. The transport protein atypical chemokine receptor 1 (ACKR1) plays a key role during inflammation in transporting chemokines from the tissue across the endothelial cells, thereby activating integrins on rolling leukocytes [57,58]. The result of the chemokine-induced inside-out signaling is a conformational change in the integrins that allows for tighter binding to cell surface ligands leading from slow rolling to firm adhesion [59]. Integrin α_4_β_1_ binding VCAM-1 or integrin α_L_β_2_ binding ICAM-1 play prominent roles during this phase [60]. Studies of the α_L_β_2_-ICAM-1 interaction have shown that blood flow stabilizes LFA-1 in the extended, high-affinity conformation and thus is important to make the arrest efficient [42,61,62]. The last step of extravasation concerns transmigration or diapedesis. During this phase, leukocytes rearrange the actin cytoskeleton to support motility on the surface of the endothelium and then squeeze their way through the spaces between neighboring EC (paracellularly) or occasionally through individual EC (transcellularly) [54,63]. Chemokine signaling plays a decisive role in this process as well, and α_L_β_2_-ICAM-1 interaction appears to be of superior importance during this step [40] (Figure 2).

During leukocyte-EC interaction LFA-1 and Mac-1 redistribute into punctuate regions at the site of contact, coinciding with actin cytoskeletal rearrangements and the appearance of ICAM clusters on the apical side of the EC [64,65,66]. At this point, the EC surface may be seen to rise and embrace an adherent leukocyte forming a so-called transmigratory cup [67] preceding both paracellular and transcellular migration. What causes the formation of this structure and the importance of it is not clear. Possibly it is important to resist shear stress exerted by blood flow, which is likely the most imminent challenge to a newly adhered leukocyte [40]. Then, during crawling, leukocytes extend dynamic membrane protrusions, rich in actin and integrins, that create invaginations onto the surface of the EC as well as invade endothelial junctions. Carman et al. proposed that these structures, called invasive protrusions, were intended to probe the EC surface to find a site permissive of the transmigration [68]. Studies indicate that leukocytes make choices on whether to embark on paracellular or transcellular migration, influenced by several factors including cellular identity [69], absence or presence of inflammatory conditions [70], the tightness of endothelial cell junctions [71], stiffness of the EC [72,73], chemokine shuttling by ACKR1 [74] and surface density of certain adhesive cell surface ligands, e.g., PECAM-1 or ICAM-1 [69,70,75,76,77]. Of note, paracellular diapedesis is preferred where three endothelial cells meet, i.e., tricellular junctions [78,79] and this was recently shown to be the case also for CD4+ effector memory cells traversing mouse brain microvascular endothelium under conditions of physiological flow [80].

In mechanistic terms, LFA-1/ICAM-1 interaction plays a prominent part in both types of transendothelial migration. In paracellular diapedesis, ICAM-1 and PECAM-1 signals work jointly to cause the initial loosening of the adherens junctions between neighboring EC by altering the phosphorylation status of key tyrosine residues in vascular-endothelial cadherin (VE-cadherin) [81,82,83]. In transcellular diapedesis, LFA-1/ICAM-1 clusters are seen preceding the formation of a transcellular pore through the EC and undergoing dynamic translocation from the apical side to the basal membrane coinciding with the sliding of a leukocyte through an EC. The pore itself is formed by F-actin and fusion of caveolae, i.e., vesicles otherwise involved in transporting molecules across cells, and the caveolae-regulator protein caveolin-1 and actin are both in contact with ICAM-1 during this process [84].

## 3. CNS Barriers and Entry Routes

The CNS is well protected behind the bone of the skull and spine, respectively, and further wrapped by the meninges and immersed in CSF. The meninges comprise four layers: the dura mater, the arachnoid mater, the pia mater and the most recently identified subarachnoid lymphatic-like membrane (SLYM) [85]. The outermost layer is the dura mater. It is directly attached to the adjacent bone tissue and is home to the superior and transversal sagittal sinuses as well as lymphatic vessels. Below is the arachnoid mater, which forms pillars of connective tissue, so-called arachnoid trabeculae, all the way down to the pia mater, creating a space between them called the subarachnoid space (SAS), which is filled by CSF. The arachnoid mater is primarily composed of collagen and fibroblasts, with the fibroblasts closest to the dural border having tight junctions between adjacent cells forming the so-called arachnoid or meningeal barrier [86]. As the blood vessels in the above dura are fenestrated, i.e., contain window-like openings between adjacent endothelial cells, the arachnoid barrier de facto becomes a barrier between blood and CSF, referred to as the arachnoid blood-CSF barrier [87]. The arachnoid barrier is impenetrable to the solutes and cells in the CSF. However, pockets of an arachnoid membrane known as arachnoid villi or granulations protrude into the dura mater and enter the large venous sinuses and at these sites drainage of CSF occurs. It should be noted that other sites of CSF drainage also exist [88].

**Figure 3:**
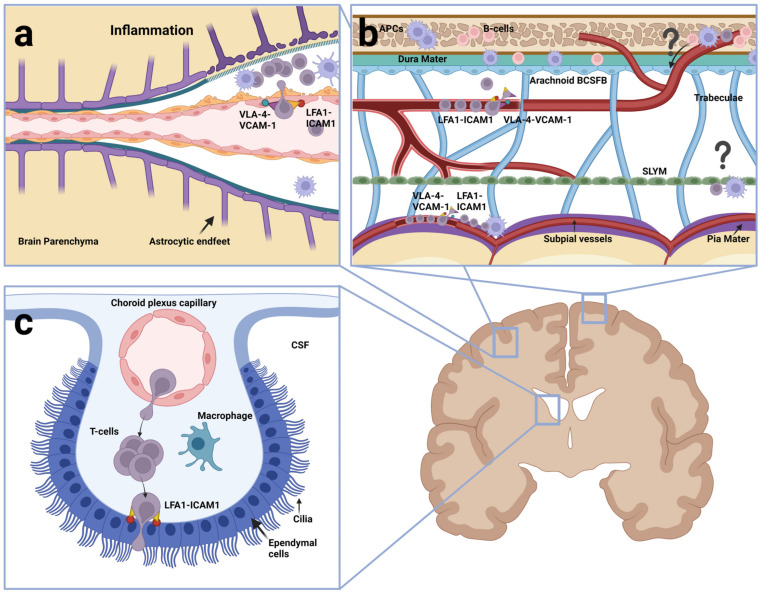
Entry routes and integrins used by normal lymphocytes (**a**) Postcapillary venule. In the presence of inflammation, the crossing of the BBB occurs in a manner dependent on VLA-4 (α_4_β_1_): VCAM-1 and LFA-1 (α_L_β_2_): ICAM-1. (**b**) Section showing meningeal layers between the calvaria and the brain parenchyma. Lymphocytes may adhere and extravasate from subpial and meningeal vessels, independently of inflammation, using VLA-4: VCAM-1 and LFA-1: ICAM-1. T cells and other immune cells may migrate from calvarial bone marrow into the dura. Whether lymphocytes migrate on emissary vessels and how they cross the arachnoid BCSFB (light blue cell layer) is not known. Whether they interact with cells of the SLYM (green) is also unknown (indicated by question marks). (**c**) Choroid plexus (CP). Lymphocytes cross the fenestrated capillaries underneath the CP. LFA-1-ICAM-1 has been shown to be involved when T cells cross the ependymal cells (blue) and enter CSF (white). This figure was created with BioRender.com.

The pia mater is the innermost of the meningeal layers and consists of flattened fibroblasts, one cell layer thick and without tight junctions. It sheaths the entire CNS including the pial arteries and some of the pial veins that run parallel to the surface of the cerebral cortex or enter or leave its surface. This arrangement creates a thin separation between any subpial spaces and the SAS above [87]. Above the pia, until recently, the SAS was thought to represent one continuous CSF-filled compartment. However, with the surprising finding of the SLYM, it is now known that the SAS is split in two, i.e., an outer superficial subarachnoid compartment and an inner deep subarachnoid compartment. The SLYM consists of loosely connected collagen intermixed with a continuous monolayer of Prox1-positive, allegedly lymphatic endothelial cells. The SLYM is conceived to enfold the arachnoid trabeculae and run around the entire CNS. Injection of fluorescent tracers showed that the compartmentalization of the SAS is likely to be complete and that the SLYM is impenetrable to molecules ≥3 kDa. Hence, tetramethylrhodamine-dextran (3 kDa) injected in the cisterna magna remained in the inner deep subarachnoid compartment [85] (Figure 3).

Historically, amongst the barriers restricting entry into the CNS, the focus has been on the blood–brain barrier (BBB) and the blood-cerebrospinal fluid barrier (BCSFB) at the choroid plexuses. The capillary endothelium is the anatomical basis of BBB, because of the tight junctions between the endothelial cells that prevent the passage of cells and macromolecules and a limited capacity for pinocytosis by the endothelial cells [89,90]. At the level of microcapillaries deep within the brain, the endothelium is part of a composite layer of different cell types and membranes including the basement membrane (BM) of the EC and its associated pericytes as well as the glia limitans. The latter is the outermost layer of the parenchyma and is made from the parenchymal BM and endfeet processes extended from astrocytes inside the parenchyma. In contrast, at the level of arterioles and postcapillary venules, a space exists between the EC BM and the glia limitans. This space, known as the perivascular or Virchow-Robin space, widens towards the surface of the brain where it fuses with the subarachnoid/leptomeningeal space [91]. The entire subarachnoid space and the perivascular space around venules and arterioles are filled with CSF (Figure 3a,b). The CSF, itself, is thought to be produced mainly by the choroid plexuses (CP), which are specialized structures situated at the lateral, third and fourth ventricles. Each of the CPs consists of part of the ependymal epithelium lining the ventricles, which have expanded into folded structures composed of ependymal cells on the outside, resting on a BM, and with a rich supply of capillaries underneath. The ependymal cells are closely connected by tight junctions and densely covered with microvilli on the apical side, whereas the endothelium beneath is fenestrated allowing free passage of molecules and cells [92] (Figure 3c). Therefore, the main barrier of the BCSFB is made from the ependymal cells as opposed to the anatomical basis of BBB, which is the endothelium. 

The CNS is considered an immunologically specialized environment. In healthy individuals, a limited number of immune cells are present, mainly myeloid cells and CD4+ memory T cells [53] and the CNS is equipped with a special lymphatic drainage system in which the CSF constitutes the functional equivalent to lymph and drains into the large venous sinuses embedded in the dura [93,94]. Tracer studies and two-photon laser scanning microscopic studies in mice have elucidated the existence of a so-called glymphatic system in which CSF enters the brain parenchyma via AQP4 water channels in the astrocytic endfeet of the glia limitans. This movement of CSF into the parenchyma leads to a rapid interchange of CSF and interstitial fluid along with a convective flux of fluid towards the perivenous spaces surrounding the large deep veins, where it is collected and drained further towards the dural sinuses [88,95]. Following the recent finding of SLYM, which compartmentalizes the SAS, it is unclear how the recently produced CSF flowing into the parenchyma is separated from the CSF containing parenchymal solutes flowing out into perivenous spaces to be drained in the dural sinuses. Allegedly, the SLYM is organized at the cerebral surface around the exit points of the large deeper veins, in a manner that effectively upholds the compartmentalization. 

Typically, three distinct routes have been considered important for leukocyte entry into the CNS, each starting from the blood and ending in the CSF. One route leads across postcapillary venules to the perivascular spaces (Figure 3a). A second route is through meningeal vessels to the subarachnoid space (Figure 3b) and a third route is from the capillaries underneath the CP and across the ependymal lining of said same (Figure 3c) [53]. With the recent discovery of direct vascular channels connecting the skull bone marrow and the meninges, at least a fourth route should be considered. It leads from the skull bone marrow into the dura mater and across the arachnoid barrier into the subarachnoid space (Figure 3b) [96,97,98]. Irrespective of the route into the CSF, the further journey into the brain parenchyma proper would require the leukocytes to cross the glia limitans, which involves traversing the parenchymal BM and close lining of astrocytic endfeet [99]. The extent to which normal immune cells use these routes and how far they go, however, is highly dependent on whether the brain is affected by inflammation and, in the case of CD4 T cells, whether these are activated.

## 4. CNS Entry of Normal Lymphocytes

As part of immune surveillance of the CNS, normal immune cells, primarily T cells, appear to cross the BCSFB at the CPs but generally remain within the CSF [17]. In addition, recent studies have shown that normal immune cells including B-cells, monocytes and neutrophils enter the dura mater along vessels connecting calvaria bone marrow with the meninges [96,98]. According to the work of Schafflick et al. (2021), approximately 2% of the leukocytes in CSF from naïve mice were shown to be B-cells, but it was not determined whether these cells had crossed the arachnoid BCSFB or the CP BCSFB [96]. Pointing to a route across the CP BCSFB, early flowcytometric studies detected activated central memory T cells (CD4+/CD45RA−/CD27+/CD69+) in the CSF from NIND (non-inflammatory neurological) patients and in parallel, CD3+ T cells were detected by immunohistochemistry in the choroid plexus stroma in autopsy CNS sections from individuals who died without neurological or inflammatory disease [100]. Other studies using immunohistochemical stainings and employing blocking antibodies in mouse experiments, provided evidence that P-selectin, but not integrin α_4_, was involved in the migration of T cells into the non-inflamed CNS [101,102]. Corroborating these studies, others reported that VCAM-1 (i.e., the surface ligand of the α_4_β_1_/VLA-4 integrin) was absent on endothelial cells underneath the CP [103] and only present on the ependymal cells on the apical side turning away from the endothelium [104]. Passage through the fenestrated endothelium is likely not to be an issue but crossing the ependymal lining may be troublesome. At present, in vitro studies have confirmed that CD4+ T cells can cross a monolayer of CP epithelial cells using ICAM-1 on the luminal side during the final step of diapedesis and release into CSF [105,106] (Figure 3c). However, direct in vivo evidence that immune cells pass either paracellularly or transcellularly through the ependymal lining is still lacking. Rather than crossing the BCSFB, it has been proposed that the immune cells migrate along the ependymal BM and leave at the base of the CP [99], where the ependymal cells possess the less restrictive gap junctions rather than tight junctions [107].

Unlike the BCSFB, the BBB of cerebral vessels in healthy brains do not allow crossing by normal immune cells [108]. In the work by Piccio et al. (2002), intravital microscopy was used to monitor whether fluorescence-labelled lymphocytes would cross parenchymal venules, following the injection into the common carotid artery in mice with ligation of the ipsilateral external carotid. The latter facilitated the entry of the injected cells into the internal carotid and further to cerebral post-capillary venules. In healthy mice, no lymphocyte-endothelial binding was observed, even when using activated neuroantigen-specific encephalitogenic lymphocytes. To sustain extravasation, the cerebral venules had to be treated beforehand with injection of lipopolysaccharide (LPS) or tumor necrosis factor alpha (TNFα) to mimic inflammation [108] (Figure 3a). Indeed, BBB endothelial cells lack the internal storage organelles containing P-selectin as well as expression of ACKR1 [109] and these molecules are expressed de novo upon systemic injection with LPS or TNFα. Alongside, the expression of both VCAM-1, ICAM-1 and E-selectin are induced [108,110,111]. Other early intravital studies using encephalitogenic activated CD4 T cells have focused on subarachnoid or subpial veins in the brain or spinal cord [112,113,114]. One study, performed using a spinal cord window to visualize CNS white matter microvasculature in SJL/N mice, showed arrest without rolling of encephalitogenic T cells. This was α_4_β_1_ and VCAM-1 dependent, but G-protein independent, suggesting no need for chemokine activation of the integrin [115]. Of note, VCAM-1 was found to be constitutively expressed on venules and upregulated during EAE in SJL/N mice used for the latter study [116]. Another intravital study of experimental autoimmune encephalomyelitis (EAE) showed intraluminal crawling, extravasation and subsequent extravascular crawling of activated T cells on subpial vessels. In this study, anti-LFA-1 and anti-VLA-4 antibodies in combination, detached crawling T cells from the luminal walls almost instantaneously [114] (Figure 3b).

Collectively, the above-mentioned intravital studies point to differences between cerebral postcapillary venules and subarachnoid/subpial vessels in terms of how well they support the adhesion of encephalitogenic T cells. In general, the effect of blocking antibodies strongly supports the role of α_4_-integrin in the arrest of lymphocytes on endothelium in EAE and in multiple sclerosis (MS) [112,114,115,116,117]. Early work showed that antibodies to α_4_ and β_1_ integrin, but not antibodies to a range of other integrins, prevented the binding of lymphocytes to brain endothelium from rats with induced EAE [118]. Later, a humanized α_4_ integrin blocking antibody, natalizumab, was shown to block the transmigration of immune cells from MS patients across layers mimicking BBB in vitro and reduce the CD4/CD8 T cell ratio in CSF from MS patients [119] as well as the relapse frequency in MS patients [120]. A subpopulation of Th17 cells, which constitutes a pathogenic T cell population in EAE, cross the CP barrier independent of α_4_-integrin [121,122]. These cells use α_L_β_2_ (LFA-1) to engage in adhesion to ICAM-1 [123]. The interaction between α_L_β_2_ (LFA-1) and ICAM-1 and -2 is believed to be important for the arrest and subsequent crawling on the endothelium cells in search of tricellular junctions that are permissive sites for diapedesis [70,80].

Once inside the CSF, the fate of the activated T cells is dependent on whether they recognize their cognate antigen on antigen presenting cells (APCs), present in the perivascular spaces or embedded in meningeal layers [114]. The work by Møllgård and coworkers (2023) showed that SLYM is home to many CD45+ cells, including both macrophages and dendritic cells, suggesting it is a major site for antigen presentation [85]. If activated T cells entering via CP, are not presented to their cognate antigens, they would be expected to undergo apoptosis, undergo differentiation to central memory T cells or tissue-resident T cells or alternatively, leave the CSF and reenter the blood [124]. Interestingly, single cell sequencing studies comparing blood and CSF immune cell profiles, have shown that T cells in CSF and blood from the same individual are rather distinct, indicating that T cells in CSF tend to stay in the CSF [125,126]. It is not known whether the CNS barriers and membranes including the SLYM are contributing to keeping immune cells from leaving the CSF and reenter circulation. Parenthetically, with the compartmentalization of the SAS by SLYM, the question of whether activated T cells in CSF remain as tissue resident T cells may be a question of whether they are present in the outer superficial or inner deep subarachnoid compartment. In turn, this could be a question of whether they originally entered the CSF across the arachnoid or CP BCSFB.

## 5. ALL Dissemination to the CNS

**Figure 4:**
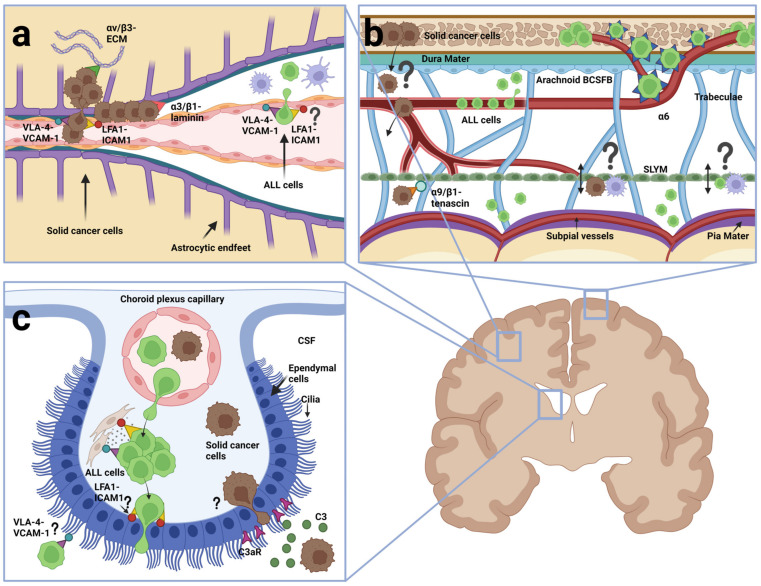
Entry routes and integrins used by ALL cells (green) and metastatic cells from solid cancers (brown) (**a**) Postcapillary venule. Metastatic cells extravasate from venules in a manner dependent on VLA-4 (α_4_β_1_): VCAM-1 and LFA-1 (α_L_β_2_)-ICAM-1. Once across, they either engage in vascular co-optive growth to which α_3_ and β_1_ integrins may contribute, or they establish metastasis in the parenchyma, which involves α_V_β3. Although presumed to involve α_4_β_1_: VCAM-1 and α_L_β_2_-ICAM-1, little is known of integrins involved in ALL extravasation from postcapillary venules. (**b**) section showing meningeal layers between the calvaria and the brain parenchyma. ALL cells use α6 integrin: laminin binding to migrate on the basement membrane of emissary vessels connecting calvarial bone marrow and the meninges. It is unknown if and how metastatic cells from solid cancers enter the meninges from calvarial bone marrow. Both ALL cells and metastatic cells are found in the dura and leptomeninges. Different integrins may be involved in the binding to meningeal components. It is unknown whether cancer cells of both types traverse or otherwise interact with the SLYM (indicated by a question mark). (**c**) choroid plexus (CP). ALL cells use α_4_β_1_: VCAM-1 and α_L_β_2_: ICAM-1 to interact with stromal fibroblasts and studies show that they can cross the BCSFB. Metastatic cancer cells in the CSF use the complement component C3 binding the receptor C3aR on the ependymal cells to disrupt the BCSFB. This figure was created with BioRender.com.

The first comprehensive study to investigate ALL dissemination to the CNS was reported by Price and Johnson in 1972. Based on histochemical examinations of brain sections from 126 autopsied leukemia patients, 55% were found to have stage 1–3 arachnoid leukemia. Only 13% had signs of a compromised pia-glial membrane with leukemic blasts having invaded the brain parenchyma (stage 3), whereas the majority of the patients had blasts associated with the arachnoid, either superficially (34%, stage 1) or deeper into the perivascular spaces between vessels and the pia-glial membrane (8%, stage 2) [127]. This high tendency for CNS involvement, including a predominance of leptomeningeal involvement, has later been confirmed in other autopsy studies [6] and in xenograft mouse models [128,129,130]. Williams et al. (2016) showed that 23 of 29 (79%) xenotransplanted BCP-ALL samples from patients resulted in CNS engraftment and serial dilution experiments showed that CNS engraftment was observed in five of six mice after transplantation of as few as ten leukemic blasts [129]. The results of histopathological examinations of the mouse brains with leukemic infiltration closely mimicked what had been observed in patients showing leukemic cells around the dural venous sinuses and within the leptomeninges but absent within the brain parenchyma [128,129,130]. Collectively, these data argue that leukemic blasts primarily enter the CSF without invasion of the brain itself, possibly signifying a capability of crossing postcapillary venules (Figure 4a), meningeal vessels (Figure 4b) and the BCSFB (Figure 4c), but not the BBB of cerebral vessels, similar to what has been reported for normal immune cells during immune surveillance in healthy brains. Indeed, one study reported the finding of leukemic cells at the choroid plexuses [129] whereas another study found no evidence of such but rather pointed to an entry along vessels from calvaria into the meninges [130]. Recent work by Rajakumar et al. (2021) used BCR-ABL1 positive, patient-derived BCP-ALL xenotransplanted into the femur of NSG mice to demonstrate that the ALL blasts invaded calvarial and vertebral bone marrow well before being identified in the SAS and that the blasts themselves created channels through RANKL-mediated bone destruction to gain access to the dura mater, arachnoid mater and finally SAS [131]. These studies may be interpreted as evidence for entry into SAS across the ependymal epithelial cells at the CP or the arachnoid barrier fibroblasts, respectively.

In vitro cell culture models mimicking the BCSFB have been developed using rodent or human epithelial cells, immortalized by SV40 large T antigen or human papilloma virus. Based on these models, it was demonstrated that BCP-ALL and T-ALL cell lines like normal T cells are capable of crossing monolayers of CP epithelial cells in response to CSF-borne chemokines [106,132,133]. Using transmission electron microcopy, März et al. (2018) found evidence of both paracellular and transcellular migration of T-ALL and BCP-ALL cells in an assay testing CXC12/SDF-1 attracted migration across a monolayer of human CP papilloma cells HIBCPP [132]. Analogous culture models testing ALL migration across BBB in general show little transmigration unless tested after endothelial activation, e.g., resulting from cytokines or exosomes emitted by leukemic blasts or treatment-related neurotoxicity [134,135]. Of note, Erb et al. (2020) recently demonstrated that exosomes from ALL cell lines could also facilitate transendothelial migration across the BCSFB, using the same HIBCPP model as März et al. (2018). The transmigration occurred without destruction of barrier integrity, pointing to exosome-facilitated transcellular transmigration, and in one of three cell lines, the T-ALL cell line P12, blocking of integrins α_5_, α_V_, β_1_ and β_3_, reduced basolateral uptake of the exosomes by HIBCPP cells [136].

The collective data above could be interpreted as evidence that ALL cells enter the CNS along ways described or speculated to exist for normal immune cells during immune surveillance of non-inflamed CNS, reviewed in the former section. Additionally, like normal T cells, ALL cells remain largely confined to the CSF. A comprehensive analysis of integrin expression in BCP-ALL cells from clinical bone marrow samples showed widespread expression of integrins including relative high expression of *ITGA4*, *ITGA5*, *ITGA6*, *ITGB1* and *ITGB7* as well as *ITGB2*, *ITGAL*, *ITGAM* and *ITGAX* genes [137]. As such, BCP-ALL appears to reflect the integrin expression profile of normal lymphocytes [32,33] making the observed similarities between ALL and lymphocytes in terms of entry routes and CSF confinement less surprising. Like normal lymphocytes, ALL cells would be able to express the integrin heterodimers α_4_β_1_ and α_4_β_7_ binding VCAM-1 and α_L_β_2_ binding ICAM-1 to engage in the extravasation from blood vessels in the leptomeninges or postcapillary venules. Once inside the CSF, they could use the same molecules to adhere to the vessels they traversed or bind ICAMs and VCAM-1 on astrocytic endfeet processes in the glial membrane [138]. Alternatively, the integrin repertoire could facilitate the adhesion to ICAMs and VCAM-1 on the apical side of CP epithelial cells. In a recent study, Fernández-Sevilla et al. (2020) showed that BCP-ALL cells from patient samples injected into the tail vein of NSG mice ended up in the stroma beneath the CP, where they made α_4_β_1_/VCAM-1 dependent contacts to the stromal fibroblasts. Interestingly, although finding foci of ALL cells in the CP, Fernández-Sevilla et al. only exceptionally observed ALL cells transit from the basal to the apical surface of the CP epithelium [139], thus on one hand confirming the presence of ALL in the CP as demonstrated by Williams et al. [129], and on the other hand, confirming the absence of CP BCSFB crossing as described by Yao et al. [130].

As described in the former section, small vessels connecting the bone marrow with the dura mater exist, which serve as entry routes for normal immune cells into the meninges [96,97,98]. In 2018, Yao et al. reported that ALL cells disseminated into the CNS taking advantage of these connecting vessels, but surprisingly migrating on the abluminal site of the endothelium using integrin α_6_ to adhere to laminin in the BM. Using this non-hematogenous route, the ALL cells bypassed the need for traversing the BBB or the choroid plexus BCSFB, as it brought them directly into the outer layers of the meninges and further across or somehow around the arachnoid BCSFB into the CSF [130] (Figure 4b). The reason why Yao et al. investigated integrin α_6_ was that the PI3Kδ inhibitor GS-649443, which reduced CNS involvement of Nalm-6 cells, also caused a reduction in ITGA6 mRNA levels. However, blocking antibodies to this integrin α_6_ reduced but did not abolish ALL cells in the CSF, which suggested that other proteins could also be important, possibly including other integrins. Scharff et al. (2019) investigated the integrin expression in a large number of bone marrow samples from BCP-ALL patients and tested for the association with CSF dissemination, finding that ITGA6 mRNA and surface α_6_/CD49f expression correlated negatively with CSF blast count. Instead, a significant association was found between blasts in CSF and ITGA5 or ITGA9 mRNA levels suggesting a role of α_5_β_1_ or α_9_β_1_ [137]. The apparent contradiction between these data with respect to integrin α6 may be due to obvious differences between xenograft mouse models and clinical samples or it may be that the work of Scharff et al. overlooked minor subpopulations of integrin α_6_ expressing cells in the bone marrow responsible for CSF involvement. Further studies are warranted to address the role of integrins, other than α_6_, in the migration of emissary vessels between bone marrow and CNS, and whether and how the arachnoid BCSFB barrier is traversed.

## 6. CNS Metastasis of Solid Cancers

Amongst solid cancers, the frequency of metastasis to the CNS is the highest in the case of non-small-cell lung cancer (NSCLC), breast cancer, and malignant melanoma [140]. The most common site of metastasis is the brain parenchyma, but metastasis in the dura mater and leptomeninges are also frequently seen. Leptomeningeal metastasis is defined as cancer infiltration of the leptomeninges, including arachnoid, SAS, and pia mater. It is seen in 5–7% of melanoma patients [141], and in 3–5% of NSCLC [142] and breast cancer patients [143]. Interestingly, the most common type of primary brain tumor in the youngest children, medulloblastoma, metastasizes to the spinal and intracranial leptomeninges but rarely outside the CNS [144].

Multiphoton laser scanning microscopy has been used to monitor the early and long-term interaction of human melanoma and lung cancer cells with cerebral microvessels following injection into the carotid artery in mice with prior ligation of the external and the common carotid artery. Both cancer types moved passively with blood until they slowed down in small microvessels with a diameter like that of the cancer cells, arrested at branch points and engaged in early extravasation across the BBB. Of note, following diapedesis, the cancer cells maintained close contact with the vessels, and in particular, melanoma engaged in perivascular growth referred to as vascular cooption (Figure 4a) [145]. The depth limitations of multiphoton microscopy prevent real-time imaging of CPs but the existence of leptomeningeal and dural metastasis suggests that these cancers also can cross the BCSFB (Figure 4b,c). Boire et al. (2017), using CSF sampling from four different leptomeningeal metastatic mouse models based on human breast and lung cancer cell lines, found that cancer cells from CSF produced the complement component C3, which they used for activation of the C3a receptor on CP epithelium to disrupt the BCSFB [146]. Furthermore, Vandenhaute et al. (2015) found that two neuroblastoma cell lines could cross an in vitro BCSFB model based on an immortalized human choroid plexus papilloma cell line (HIBCCP), but without compromising barrier integrity [147]. This finding indicates that at least some human solid cancers might possess the capabilities to cross the BCSFB in vivo (Figure 4c). However, the involvement of integrins and integrin ligands was not addressed.

It is believed that integrins and chemokine signaling contribute to explaining the observed organ-specific metastasis of solid cancers providing present-day proof of Paget’s “seed and soil” hypothesis formulated more than a hundred years ago [148]. Hence, chemokine receptors and integrins fit the description of properties of the cancer cells, i.e., “the seeds” whereas chemokines and integrin ligands represent properties of the organ, i.e., the “soil”, which must be compatible for homing, seeding, survival and growth of metastatic cells. A good example is the CCR9-mediated metastasis of a subset of melanoma cells to the intestine due to the expression of CCL25 there [149], which is intriguing considering the normal role of CCR9:CCL25 in the recruitment of CCR9+ T cells via activation of α_4_β_7_ [41,150]. Several studies argue that CXCR4:CXCL12 plays an analogous, decisive role in breast and lung cancer metastasis to the CNS [151,152,153]. With respect to integrin-dependent adhesion and extravasation from brain vessels, data confirm the expected importance of VCAM-1 and ICAM-1. In the case of melanoma, the endothelial expression levels of VCAM-1 were shown to correlate with metastatic pattern [154]. For decades, VLA-4 (α_4_β_1_) has been a known marker of melanoma cells, and its role in binding to VCAM-1 on EC and metastasis is well documented, but the majority of these studies focused on lung metastasis and were based on ECs from outside the CNS [155,156,157,158,159,160]. Importantly, Garcia-Martin et al. (2019) showed that 92% of all human melanoma brain metastases stained positive for VLA-4 in tissue microarrays, and an anti-α_4_ integrin antibody blocked melanoma intercalation into layers of primary mouse brain microvascular EC [161]. In a lung cancer model, the enhancement of brain metastasis by inflammatory conditions was shown to involve VCAM-1 and ICAM-1 on brain endothelium, but inflammation did not enhance lung cancer cell adhesion to and extravasation from brain vessels or brain metastasis in *icam-1* deficient mice. Furthermore, a VCAM-1 neutralizing antibody prevented the adhesion of the lung cancer cells to cultured brain EC [162]. Using mouse 4T1 and human MDA-231BR breast cancer models, Soto et al. (2013) demonstrated an increase in many cell adhesion molecules (CAMs) on the surface of brain endothelial cells, including the Ig superfamily CAMs, VCAM-1 and ICAM-1, as well as VLA-4 and integrin β_4_ early during breast cancer colonization of the brain. In parallel, the cancer cells were demonstrated to express the same CAMs, and an anti-α_4_ blocking antibody caused a significant reduction in the number of metastatic colonies of MDA-231BR cells confirming its alleged role in adhesion to EC [163]. The cancer-induced increase of integrin expression in EC as well as the reciprocal expression of the integrin ligands, VCAM-1 and ICAM-1, on the surface of the cancer cells is the opposite to what is described for the interactions between EC and ALL cells as well as EC and normal lymphocytes. This may signify, that not all the roles of VCAM-1 and ICAM-1 in solid cancer metastasis necessarily implicate integrins. Hence, Ig superfamily CAMs are capable of homophilic and heterophilic interaction, and recent work demonstrated the role of homophilic ICAM-1-ICAM-1 interaction in the formation of homotypic tumor cell clusters and lung metastasis of breast cancer cells [164]. 

In addition to the role of VCAM-1 on the EC of the BBB, a role of VCAM-1 was proposed to exist inside the leptomeninges, which allegedly depended on α_4_β_1_. The binding of B16F-10 murine melanoma cells to mouse leptomeningeal cells could be blocked by VCAM-1 antibodies with no additive effect of blocking integrin β_1_ and β_3_ [165]. Of note, the binding of medulloblastoma cells to leptomeningeal ECM proteins suggested that other integrins involving α_V_, β_1_ and β_3_ may be important [166], specifically α_9_β_1_ binding tenascin [167]. Much of the later work has dealt with α_V_β_3_ showing a role of this integrin in angiogenesis or growth inside the brain parenchyma. Overexpression of activated α_V_β_3_ mutant in the MDA-MB-435 breast cancer cell line increased brain metastasis by way of VEGF upregulation and increased angiogenesis [168], whereas an anti-integrin α_V_ monoclonal antibody reduced brain metastasis of MDA-MB-231-breast cancer cells overexpressing the HER2 oncogene [169]. Additionally, the α_V_β_3_ receptor antagonist cilengitide was shown to suppress melanoma cells growing as xenotransplants in the forebrain of nude mice [170] and Küsters et al. (2001) reported that two α_V_β_3_ expressing melanoma cell lines exclusively produced metastasis in the brain parenchyma, whereas two cell lines, devoid of α_V_β_3_, preferentially metastasized to dura mater and leptomeninges [171]. In the latter study, transfection of integrin β_3_ into non-expressing melanoma cells did not alter their site of metastasis, which could be due to insufficiently activated α_V_β_3_ by this approach or indicate that α_V_β_3_ is not sufficient to generate brain metastasis. It should be noted that to cross the BBB, metastatic cells are known to employ a range of genes, including proteases [172,173,174,175].

The first host cells that metastatic cancer cells encounter when attempting to extravasate are endothelial cells. Due to the finding of metastatic cells growing on the exterior of pre-existing blood vessels of the CNS, Carbonell et al. (2009) tested whether the vascular BM rather than the neuronal compartment should rightfully be considered the “soil” of the brain to which metastatic cells were homing. Using a combination of in vitro adhesion assays and experimental brain metastasis assays, they found that breast cancer cell lines used β1 integrin to adhere to the vasculature BM. Importantly, blocking the β_1_ integrin subunit on the cancer cells prevented adhesion to vascular BM and attenuated both metastasis establishment and growth in vivo, indeed supporting the concept of the vascular soil [176]. Not addressed in this work, was the identity of the α-subunit binding β_1_ and the ligand of the alleged integrin heterodimer within the BM, e.g., laminin, collagen or fibronectin. In the case of highly metastatic NSCLC cells, binding to laminin and brain metastasis have been associated with integrin α_3_β_1_ [177]. Furthermore, other β integrins may play an indirect role in the interaction with the vascular “soil” without participation in the actual binding. Hence, integrin β_4_ signaling was shown to promote breast cancer cell adhesion to brain microvascular endothelium through induction of ErbB2-mediated secretion of VEGF [178]. Cancer cells may use blood vessels as migration tracks and sites of growth in the leptomeninges in order not to get stranded in the CSF, which is low in metabolic intermediates and micronutrients as well as markedly hypoxic [179]. Of note, the cooperation of VEGF and integrin signaling is likely of prime importance for vascular cooption by cancer cells in a hypoxic microenvironment. Hypoxia is a known inducer of VEGF-A [180], which in turn increases the permeability of the endothelium to both molecules and cells [181] as well as increases cellular adhesion to ECs via upregulation of ICAM-1 and VCAM-1 [182,183]. Of particular interest, VEGF-A and integrin α_6_β_1_ are known to cooperate in the formation of a perivascular niche for cancer stem cell (CSC) expansion in the case of breast cancer [184,185,186]. In ALL, the concept of vascular cooption involving conjoint VEGF-A and α_6_β_1_ signaling is highly intriguing considering the integrin α_6_-mediated dissemination of ALL to the CNS along the surface of microvessels [130]. Indeed, VEGF-A expression is upregulated in ALL cells in the CSF as a result of adaptation to hypoxia [187] and CSF levels of VEGF-A is a good predictor of CNS involvement [188]. Furthermore, VEGF-A is important for transendothelial migration and leptomeningeal infiltration by ALL cells [128] as well as disruption of BBB integrity during leukemic invasion of the brain parenchyma [134].

## 7. ALL Seen from a Metastasis Perspective

The inefficiency of metastasis [18,19,189,190] is in striking contrast to the high propensity of ALL to disseminate to the CNS both in patients and PDX models [26,129]. Although similarities exist between brain metastasis and CNS dissemination of ALL in terms of CNS entry routes and integrins used, differences also exist, as reviewed above and summarized in Table 1. Thus, the question remains whether the processes are de facto similar in a broader perspective. In the final section of this review, we address some of the hallmarks of metastasis and discuss whether ALL abide by these hallmarks and the implication of integrins in these contexts.

***Clonal origin and cancer stem cells***. Today, the inefficiency and phenotypic instability of metastasis are believed best explained by an updated cancer stem cell (CSC) model according to which CSCs are the cause of metastasis and their stemness is a dynamic cell trait, gained or lost due to mutations in conjunction with factors in the microenvironment. In epithelial cancers, the acquisition of stemness and metastatic capability is tightly linked to the generation of epithelial-mesenchymal hybrids during epithelial-to-mesenchymal transition (EMT), which remain in a transitory state for maximal cellular plasticity [22,23]. Although genetic alterations can be detected specific for metastatic cells compared to their respective primary tumors, these alterations are not consistently detected across patients [192,193,194,195]. The genetic alteration most consistently associated with metastasis appears to be whole genome doubling [196] and experimental models selecting for increased metastasis show that mutated oncogenes driving tumor growth, such as mutated *KRAS* and *BRAF* genes, also increase metastatic competence when amplified [197]. Hence, no universal metastasis driver mutations have been identified. Rather, metastatic cells may simply result from the amplification of the same genetically activated oncogenic pathways that drive early cancer development mixed with physiological programs from stem cell and developmental biology [198].

As in other cancers, both BCP-ALL and T-ALL carry structural chromosomal alterations that occur with relatively high frequency and allow the division of these cancers into subgroups with different diagnostic and therapeutic implications. To facilitate the discussion of the clonal origin of CNS dissemination in ALL, the genetics of ALL can be summarized as follows (for in-depth review we recommend [199,200]). In T-ALL, different subtypes are characterized by sequence mutations or copy number alterations in, e.g., *NOTCH1* or *FBXW7* as well as subtypes showing deregulated expression of genes encoding specific transcription factors (e.g., *TAL1*, *LMO1*, *LMO2*, *TLX3* and MYC), generally due to chromosomal deletions [200]. In BCP-ALL, common genetic subgroups include ALL with high hyperploidy (a minimum gain of five chromosomes), hypoploidy (less than 44 chromosomes), t(1;19) encoding E2A-PBX1, t(12;21) encoding ETV6-RUNX1, ALL with rearranged *KMT2A* (*MLL*) to a range of partners, t(9;22) or Philadelphia-chromosome positive (Ph+) ALL encoding BCR-ABL1 and a subtype called Ph-like ALL having similar gene expression profile as Ph+ ALL but no BCR-ABL1. In the latter, a major subset of cases has chromosomal rearrangements resulting in CRLF2 overexpression [199]. Typically, chromosomal rearrangements encoding fusion proteins can be considered initiating or driver mutations. In addition, secondary DNA deletions, gains or point mutations are found with varying frequency. These lesions target genes encoding lymphoid transcriptions factors (e.g., *IKZF1*, *PAX5*, *EBF1* in BCP-ALL and, e.g., *RUNX1*, *ETV6*, *GATA3* in T-ALL), controlling cell-cycle regulation (*CDKN2A*/*CDKN2B*, *RB1*), apoptosis (*ETV6*, *ERG*) or intracellular signaling pathways, e.g., via mutations in *JAK1*, *JAK3*, *STAT5B*, *KRAS*, *NF1*, *AKT1*, *PTEN* or *PTPN2* [199,200,201].

Dissemination to the CNS is associated with high-risk characteristics including T-ALL per se [11] and specific chromosomal aberrations in BCP-ALL, such as *KMT2A*, *BCR-ABL1* and *TCF3-PBX1* gene rearrangements [16,202,203,204]. The limited absolute numbers of especially T-ALL, and concomitantly low number of CNS relapses, hinders studies attempting to link cytogenetics and CNS relapse. The inadequate sensitivity of the current CNS staging method is another major limitation in clinical studies [9]. Animal experiments including PDX models have provided evidence that BCP-ALL cells harboring *BCR-ABL1* or *TCF3-PBX1* gene fusions or a range of other chromosomal rearrangements can infiltrate the CNS [129,205,206,207]. In T-ALL, *Notch1* mutations driving chemokine receptor CCR7 expression have been reported to increase CNS involvement [208]. Some authors have used these animal models as surrogate models to test for clonal evolution of CNS involvement using lentivirus integration sites or IgH VDJ rearrangements as clonality markers. The results have been interpreted in favor of a non-clonal origin of ALL dissemination to the CNS [129,209]. However, as some of these models were efficiently infiltrating the CNS beforehand, their use in clonal evolution studies selecting for CNS involvement is questionable. Other concerns deal with the appropriateness of the used clonality markers and the mouse-based models to follow the clonal selection of human cells. In opposition to these findings, another study based on gene expression profiling of clinical samples and flow cytometry pointed towards CNS involvement being associated with selected traits in the form of a particular protein signature [14], whereas functional investigations of selected genes have linked CNS involvement with genes encoding ZAP70 [210], CD79a/Igα [211], IL7-R [212], IL-15 [213,214], VEGF [128] and integrin α_6_ [130]. At present, it remains unresolved whether CNS involvement of ALL requires clonal selection of cells with special traits.

BCR-ABL1 (Ph+) and Ph-like ALLs present an interesting biology when viewed from a metastasis perspective. The lymphoid transcription factor IKZF1, commonly referred to as Ikaros, is frequently deleted in BCP-ALL [199], and in BCR-ABL1 (Ph+) and Ph-like ALLs, *IKZF1* deletions and mutations are particularly frequent, present in almost 85% [215] and 70%, respectively [201,216]. During development, *IKZF1* is turned on as hematopoietic stem cells (HSCs) develop into lymphoid multipotent progenitors (LMPPs) and is critical for the early differentiation into lymphoid-restricted progenitors [217,218]. Comparisons of global gene expression profiles of HSC and LMPP populations from Ikaros-null and wildtype mice argue that Ikaros turns on a transcriptional network, characteristic of lymphoid differentiation, and in parallel turns off transcription of genes found in the HSC signature, including genes involved in self-renewal [219]. Thus, the result of combining an *IKZF1* loss of function mutation with the *BCR-ABL1* oncogenic driver in Ph+ ALL or alternatively, amplified *CRLF2* gene expression and *JAK* mutations in Ph-like ALL, is likely leukemic cells, which retain some self-renewal capability. 

The key stem cell marker, CD34, is encoded by one of the genes downregulated by Ikaros in Ph+ BCP-ALL [220]. In human acute myeloid leukemia, the existence of leukemia stem cells (LSC) is well-established and shown to be phenotypically restricted to CD34+CD38-cells. In contrast, ALL cells at different maturation stages possess stem cell properties [221,222], not restricted to the CD34+CD38-fraction [223,224]. Despite not finding an overall difference between CD34+ and CD34-BCP-ALL samples with respect to HSC-associated genes, an enrichment of HSC genes was found in CD34+ BCP-ALL samples using a rank-based gene set enrichment analysis [225]. In this work, integrin α_6_ was among the most upregulated genes in CD34+ BCP-ALL. This integrin represents one of the most consistent stem cell markers [226,227,228], which is also a common marker in cancer stem cells in solid cancers [229,230,231] in addition to being associated with poor therapy response of BCP-ALL [137] and ALL dissemination to the CNS [130]. The upregulation of integrins and concomitant increase in adhesion to ECM has been described by a series of studies, e.g., linking increased expression of integrin α_5_ and α_6_ to BCR-ABL1 driven ALL [205,232] or linking increased expression of integrin α_5_ to Ikaros mutations in BCR-ABL1 ALL [206,233]. Of note, integrin α_4_, α_5_ and α_6_ mediate survival and chemoresistance in Ph+ ALL [234,235,236]. Rather than viewing integrins as stem cell markers per se, it has been proposed that integrins are turned on at a pre-B stage characterized by proliferation and stroma-dependency, which is followed in normal B cell development by a stroma-independent phase due to Ikaros-driven differentiation. However, when Ikaros is lost as in Ph+ and Ph-like BCP-ALL, the cancer cells remain in a non-differentiated and stroma-dependent, i.e., integrin-dependent state [217,237].

Collectively, the above-mentioned studies support the notion that Ph+ and Ph-like BCP-ALL exist in a precursor or dedifferentiated state, with some characteristics shared with HSC. The mutations in genes encoding transcription factors for early lymphoid development (*IKZF1*, *IKZF3*, *EBF1*, *PAX5*) occurring commonly in BCP-ALL across cytogenetic types [199,201], suggest a common need to remain in a dedifferentiated state. Conversely, the findings that CD79a/Igα, IL7-R and IL-15 expression each associate with CNS involvement [211,212,213,214] emphasize the need of BCP-ALL to also retain certain differentiation characteristics to disseminate to the CNS. Though speculative, these findings may indicate that ALL cells must remain in a hybrid state to disseminate to the CNS, like metastatic adenocarcinoma cells harboring oncogenic driver mutations and existing in an EMT hybrid state providing them with cellular plasticity. 

***Inflammation, exosomes and tumor-associated macrophages in the metastatic niche***. It is becoming increasingly clear that the interplay between different cell types in the tumor microenvironment (TME) is a prerequisite for metastasis and that stromal fibroblasts, ECs and tumor-associated macrophages (TAMs) are actively communicating with cancer cells in establishing a metastatic niche [238,239]. Of note, as a preluding stage to the actual metastatic niche, a premetastatic niche may exist involving the activation of the vascular endothelium in the site of future metastasis [240]. In a pioneering study, Kaplan et al. (2005) showed that lung metastasis by B16 melanoma was preceded by increased production of fibronectin by resident fibroblast-like stromal cells followed by α_4_β_1_ dependent adhesion of VEGFR1+ (CD133+, CD117+, CD34+) hematopoietic bone marrow myeloid progenitors. In the resulting premetastatic niche consisting of VEGFR1+ progenitors, fibroblasts and fibronectin, expression of CXCL12 became highly expressed leading to the homing of CXCR4+ melanoma cells and metastasis. Of note, the initial fibronectin expression by lung fibroblasts was dependent on factors secreted by the melanoma cells, but the identity of these factors was unknown at the time [241]. Later, a bulk of evidence has shown that cancer-derived exosomes account for an important part of the cancer-stroma interaction via a functional cargo of mRNA [242], miRNA [243,244], cytokines [134,245], adhesion molecules [246], growth factors [247] as well as a range of other molecules [248,249]. Exosomes from the breast cancer cell line MDA-MB-231 were shown to determine organotropic metastasis via integrin-dependent uptake in organ-specific stromal cells. Exosomes containing integrin α_6_β_4_ and α_6_β_1_ were associated with lung metastasis, while exosomal integrin α_V_β_5_ was linked to liver metastasis and notably, exosomes from lung-metastatic cancer cells could redirect metastasis to the lungs from cancer cells, otherwise shown to be liver-metastatic [246]. In this study, expressions of integrin β_3_, β_1_, α_V_, α_2_ and α_6_ were most frequently observed in exosomes from four brain-tropic breast and melanoma cell lines, but the ability of individual heterodimers in determining brain-tropic metastasis was not addressed. 

Numerous studies have addressed the role of exosomes in the ALL-stroma interaction [250,251,252]. Of note, adult T cell leukemia/lymphoma exosomes containing miRNAs, VEGF and the oncoprotein Tax alter the behavior of mesenchymal stem cells [252], whereas ALL exosomes containing various miRNAs play a role in the suppression of Th17 cells [253] and in the development of T-ALL cooperating with NOTCH1-signaling [254,255,256]. However, only a few studies have addressed the role of exosomes in leukemic dissemination to CNS. In ALL patients, fractions of CSF have been found to contain extracellular vesicles (EVs) carrying miRNAs, and notably, the miR-181a expression was found to be 52x higher in CSF from CNS + ALL patients compared to CNS-cases [257]. Furthermore, exosomes containing IL-15 were shown to be taken up by brain ECs and functionally implicated in full-blown brain metastasis of Nalm-6 BCP-ALL leukemia cells in mice [134], whereas exosomes from ALL cell lines facilitated transendothelial migration across a BCSFB model based on immortalized CP epithelial cells. In the latter report, integrins α_5_, α_V_, β_1_ and β_3_ were implicated in the exosome uptake mechanism, specifically in the T-ALL cell line P12 [136]. These data suggest that exosomal interleukins and integrins may play a part in crossing CNS barriers, possibly in advance of the ALL cells, but whether they serve to establish premetastatic niches is unknown. 

As the metastatic niche is forming, the role of TAMs and inflammatory cytokines are believed to become important. Two polarized extremes of macrophages are known to exist, i.e., M1 with proinflammatory activity and M2 with anti-inflammatory activity. The M1 type secretes Il-1β, IL-6 and TNF-α, whereas IL-4, IL-10 and IL-13 stimulate the development of the M2 type, which then produces IL-10, IL-13 and TGF-β [258]. TAMs in the TME are typically of a M2-like phenotype [259], but proinflammatory cytokines secreted from tumor-infiltrating M1 TAMs may cause increased metastasis, converting to M2 TAMs in the process. Hence, studies show that IL-6 may help sustain cancer stem cell populations and increase EMT [260,261], whereas IL-4, CCL-2 and exosomal miRNA from cancer cells cause M2 polarization of TAMs [262,263,264].

**Figure 5:**
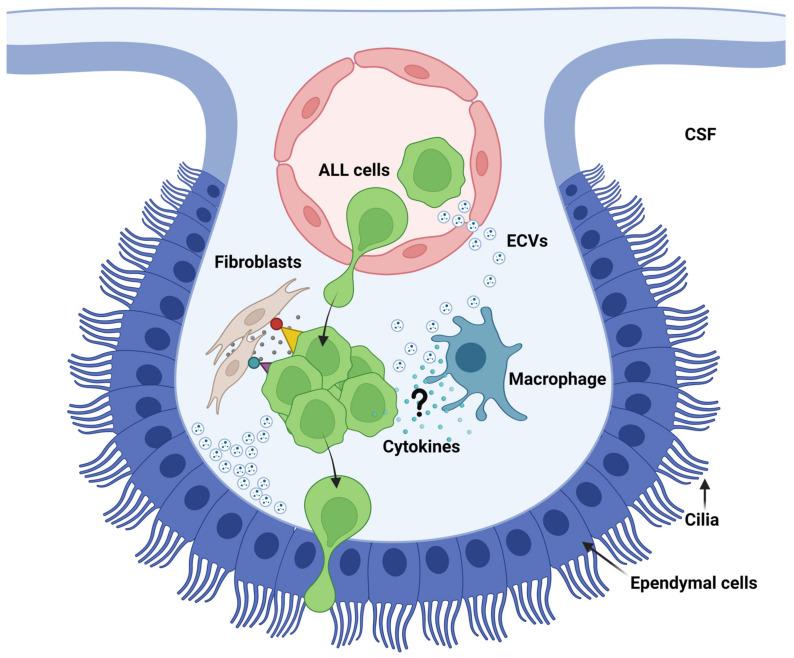
Model of ALL interaction with stromal cells of the choroid plexus. ALL cells have been shown to bind stromal fibroblasts through α_4_β_1_: VCAM-1 and α_L_β_2_: ICAM-1 converting them to cancer-associated fibroblasts and instructing them to secrete cytokines in the process. The result is increased chemoresistance of the ALL cells. Furthermore, in vitro studies have shown that extracellular vesicles produced by ALL cells can prepare ependymal cells for ALL transmigration. The role of integrins in this process has been shown for some ALL cells. It remains to be shown whether communication takes place in the CP stroma between macrophages and ALL cells, whether cytokines and exosomal integrins are involved, and whether the macrophages are converted to tumor-associated macrophages during such a process (indicated by question mark). See text for further details. This figure was created with BioRender.com.

In the CNS, macrophages comprise microglia inside the parenchyma and border-associated macrophages (BAMs) residing in the meninges, the choroid plexus and the perivascular spaces [265,266]. BAMs are strongly positive for CD206, and BAMs have also been identified as part of the SLYM as seen by existence of CD206+ CD68+ cells [85]. At present, brain macrophages are known players in brain metastasis [267], whereas there is a complete lack of studies addressing the communication between macrophages and ALL cells inside CNS niches. This is curious considering the many known responses of ALL to inflammatory cytokines in the CSF [134,212,213,214,268]. Fernández-Sevilla et al. (2020), studying the α_4_β_1_/VCAM-1 dependent interaction of ALL cells and fibroblasts within the stroma underlying the CP, found that the ALL cells gained resistance to chemotherapeutical drugs on account of the interaction whereas the fibroblasts gained cancer-associated fibroblast (CAF) markers and secreted proinflammatory markers IL-2, IL-8 and CCL2 [139]. Of note, some of these markers have been associated with CAF-induced chemoresistance by sustaining stemness in lung and breast cancer [269]. The work of Fernández-Sevilla et al. is unique in showing that the integrin-dependent interaction between ALL cells and stromal cells instructs the stromal cells to secrete cytokines that may help sustain the properties of the ALL cells. As such, it comes close to showing that ALL dissemination to the CNS conforms to a known hallmark of metastasis. BAMs are in close vicinity to ALL cells when they enter the CNS, e.g., BAMs are inside the stroma of the choroid plexus and part of the meningeal layers, including the SLYM. Whether inflammatory cytokines and macrophages contribute to creating metastatic niches at these sites is an intriguing avenue for future ALL research (Figure 5).

## 8. Clinical Perspectives

As mentioned in the introduction, a call has been made for a paradigm shift in the treatment of CNS involvement in leukemia, which includes more robust biomarkers and novel therapeutical targets [9]. Based on the examination of clinical samples, integrin α_5_, α_6_ and α_9_ stand out as the most likely candidates for biomarkers of leptomeningeal infiltration [130,137] but except for α_6_, the knowledge supporting the actual roles of these integrins in CNS involvement is lacking. Novel therapeutical strategies have focused on eradicating ALL in the bone marrow and blood by mobilizing the ALL from the bone marrow niche thus making the ALL cells vulnerable to chemotherapy. Some of these strategies have entered clinical trials, e.g., therapy based on the use of the CXCR4 antagonist AMD3100 (recently reviewed by Whiteley et al. [26]), whereas others have shown promising effects in animal models, e.g., the anti-integrin α_4_ blocking antibody natalizumab [236] and blocking antibodies to integrin α_5_ and α_6_ [130,235]. Focusing on ALL in the CNS, recent data from animal models show the therapeutical effects of CXCR4 antagonists [131,270], the anti-VEGF antibody bevacizumab [128] as well as a monoclonal antibody to integrin α_6_ [130]. Although a role for integrin α_4_ has not been firmly established in CNS ALL, natalizumab is also an attractive candidate to test in the treatment of ALL in the CNS, as it is known to prevent immune cell entry into the CNS in multiple sclerosis [120]. Given the different entry routes into the CNS and the overlapping roles of integrins, a combination of blocking antibodies to different integrins, chemokine receptor antagonists and molecules targeting VEGF/VEGFR-2 signaling will likely be the most effective approach to treat ALL in the CNS when used together with chemotherapy. Alternatively, targeting signaling pathways downstream of integrins by inhibiting PI-3-kinase or the focal adhesion kinase (FAK) may be efficient [130,235].

## 9. Conclusions

Here, we reviewed the entry routes and role of integrins in CNS surveillance by normal lymphocytes, as well as in the dissemination of ALL and the metastasis of solid cancers to the CNS. A popular perception is that ALL cells are efficiently disseminating to the CSF because they mimic normal lymphocyte surveillance mechanisms. Indeed, the literature highlights the same entry routes into the CNS for both ALL cells and normal lymphocytes. Yet, looking at integrins and integrin ligands there are not enough functional experiments reported to conclude that the same integrins and integrin ligands are important in both cell types. Studies of ALL and lymphocytes have tended to focus on different aspects, leading to findings that cannot be generalized. Of note, other lymphoid cancers such as diffuse large B-cell lymphoma (DLBCL) also display CNS involvement, albeit with a lower frequency than ALL [271,272]. The exact CNS entry mechanisms and the role of integrins are, however, only scarcely studied in DLBCL [273] and so a comparison with ALL must await further studies. 

Another recurring theme in the literature, is the alleged similarities between ALL dissemination and metastasis of solid cancer, raising the question whether the same adhesion molecules such as integrins are active when the dissemination/metastasis involves the CNS. In the case of ALL, the frequency of leptomeningeal dissemination is high whereas invasion of the brain parenchyma is seen in only around 10% of the patients. The opposite pattern is seen in the case of malignant melanoma, lung and breast cancer, where leptomeningeal metastasis occurs in less than 10% of the patients. Although both types of cancers enter the brain across the CP BCSFB, the role of integrins has only been described in the case of ALL cells in the CP stroma. Conversely, the adhesion and extravasation from cerebral vessels during brain metastasis is well studied from the point of integrins, but far less is known of integrins in ALL disseminating into the parenchyma.

Collectively, despite decades of investigations, large gaps exist in the understanding of integrins involved in the dissemination/metastasis to the CNS. One of the open questions is whether metastatic cells from solid cancers enter the CSF from calvarial bone marrow by crawling on the outside of emissary vessels, as recently shown for ALL cells using integrin α_6_-laminin dependent migration. Another open question is how both types of cancers interact with the recently identified SLYM membrane, containing macrophages and separating the CSF into compartments, and whether integrin signaling plays a role herein.

At the end of this review, we asked the fundamental question whether ALL dissemination to the CNS abides by known hallmarks of metastasis, including the importance of preserving cellular plasticity by balancing stemness and differentiation as well as the role played by inflammation and communication with stromal ECs, fibroblasts and macrophages in the establishment of the metastatic niche. Perhaps best illustrated in the case of Ph+ and Ph-like ALL harboring ikaros mutations, the dissemination of ALL may result from balancing stemness and differentiation, and integrins appear to play a central role in preserving viability during the process. Additionally, cytokines and exosomal integrins are likely playing a part in the establishment of CNS niches, analogous to what is seen in other cancers. Future research into these areas is likely to hold the answer to whether ALL dissemination to the CNS is a bona fide metastatic process. Amongst the integrins, new markers of CNS dissemination and therapeutical targets may be identified because of this work.

## Figures and Tables

**Table 1:** Summary of CNS entry routes and integrins used by normal lymphocytes, ALL cells and metastatic cells from solid cancers.

Type	Entry Route	Occurrence	Integrins and Integrin-Ligands	Selected References
normal lymphocytes	BCSFB (arachnoid)	Likely *	n.d.	[96]
	BCSFB (choroid plexus)	occurs	α_4_ and VCAM-1 independent (capillary crossing)	[100,101,102,103,104,106]
			α_L_β_2_: ICAM-1 (diapedesis across ependymal cells)	[105,122,123]
	BBB (cerebral)	occurs, if EC is activated	α_4_β_1_: VCAM1, α_L_β_2_: ICAM-1 (rolling and arrest)	[108,117,119]
	BBB (meningeal)	occurs without need for EC activation	α_4_β_1_ (EC binding) ICAM-1 (diapedesis)	[70,112,113,114,115,116,118,119]
ALL cells	BCSFB (arachnoid)	occurs	α_6_ (binding laminin in BM of emissary vessels)	[130,131]
	BCSFB (choroid plexus)	occurs	α_4_β_1_, α_L_β_2_ (adhesion to stromal fibroblasts)	[129,132,133,136,139]
			α_5_, α_V_, β_1_ and β_3_ (crossing ependymal layer)	
	BBB (cerebral)	Likely (ALL cells in parenchyma)	n.d.	[6,127,134]
	BBB (meningeal)	Likely (ALL cells in leptomeninges *)	n.d.	[127,128,129,139]
Metastatic cells from solid cancers	BCSFB (arachnoid)	Likely (metastatic cells in the SAS *)	n.d.	[141,142,143,144,191]
	BCSFB (choroid plexus)	occurs	n.d.	[146,147]
	BBB (cerebral)	occurs	VCAM-1: α_4_β_1_ and ICAM-1 (adhesion and extravasation)	[154,161,162,163,164,168,169,170,171,176,177,178]
			α_V_β_3_ (angiogenesis and growth)	
			α_3_, and β_1_ (binding to laminin in EC BM)	
	BBB (meningeal)	Likely (metastatic cells in the SAS *)	α_4_, α_V_, α_9_, β_1_ and β_3_ (adhesion within leptomeninges)	[165,166,167]

* at present, the entry route is not distinguishable from entry across choroid plexus BCSFB, n.d., not determined.
